# How to make carer involvement in mental health inpatient units happen: a focus group study with patients, carers and clinicians

**DOI:** 10.1186/s12888-017-1259-5

**Published:** 2017-03-21

**Authors:** Domenico Giacco, Aysegul Dirik, Justina Kaselionyte, Stefan Priebe

**Affiliations:** 1 Unit for Social and Community Psychiatry, (WHO Centre for Mental Health Service Development), Queen Mary University of London, E138SP, London, UK; 2 Newham Centre for Mental Health, E13 8SP, London, UK

**Keywords:** Carers, Family, Friends, Inpatient care, Psychosis, Severe mental disorders

## Abstract

**Background:**

Carers are family members or friends who support people with a mental health problem without being paid. Carer involvement in mental health treatment has been consistently supported by research evidence and promoted by policies but its implementation rates are poor. Particularly when patients are treated in inpatient units, carers often report being left without information or being excluded from decisions about treatment. In this study we have explored, along with staff perspectives, views of patients and carers who had a recent experience of inpatient mental health care on how to improve the implementation of carer involvement in inpatient care.

**Methods:**

Sixteen focus groups were held with carers, patients and clinicians in London, United Kingdom. We included staff working in inpatient units and patients and carers who had experience of inpatient care in the last five years. Data from focus groups were analysed using thematic analysis.

**Results:**

Eighty six participants in total (31 service users, 22 carers and 33 clinicians) attended the focus groups. Participants identified that generally, carer involvement should happen as soon as possible after admission, although this may be challenging in some cases. Carer involvement should include receiving information, participating in decisions about care and discharge and receiving emotional support by staff. When carers are involved, their personal knowledge of the patient’s condition should be utilised. Challenges to carer involvement may include problems with identifying carers during a mental health crisis, obtaining valid patient consent, sharing appropriate information, and contacting and engaging carers. Additionally, it was perceived that all the ward staff need to be actively engaged in order to make carer involvement happen and this cannot be left only to specifically trained clinicians.

**Conclusions:**

These findings identify basic components that all family interventions in inpatient units should have. Further studies are needed to explore how and if purposively designed clinical interventions can improve carer involvement in inpatient treatment and, consequently, patient outcomes.

## Background

“Carer” is an increasingly used term to describe people who are family members or friends of people with health conditions and provide support to them without being paid [1, 2]. In mental health care, carers can help patients and clinicians recognise and respond to early warning signs of relapse [3] and to engage patients with their care plans [4–6]. When carers are involved in treatment, patients are less likely to need frequent inpatient admissions and are more likely to experience significant improvements in their symptoms and quality of life [7–10] and family interventions have shown effectiveness for reducing relapse in severe mental disorders [11, 12]. On these grounds, mental health policies around the world consistently recommend carer involvement in the treatment of people with severe mental illness [13–17].

However, the implementation of carer involvement in clinical practice remains an unfulfilled aim of many services, and carers frequently report feeling excluded from the treatment process [18–21]. In the community care, carers reported lack of involvement in care planning and critical issues identified were structural barriers (timing and location of meetings), cultural barriers relating to power imbalances and specific barriers relating to confidentiality [22].

Carer involvement seems to be even more challenging in inpatient settings. The lack of appropriate information and involvement in clinical decisions makes it difficult for carers to resume their provision of support to the patient after discharge [20, 23–25].

In order to work well, carer involvement in treatment requires the engagement of three parties, i.e. the patients, the carers and the clinicians. However, existing studies which have explored how to facilitate carer involvement have mainly focused on clinician opinions and clinician-led models of carer involvement [19, 25–27]. As a result, there is a lack of patient and carer perspectives in the literature, with only one previous study which explored the perspectives of all three groups without focusing specifically on inpatient care [28].

In this study, we aimed to assess the perspectives of all these three parties, i.e. patients, carers and mental health clinicians on how to improve carer involvement in inpatient settings.

Firstly, we aimed to collect the views of different stakeholder groups on what carer involvement should entail as previous studies showed that the exact meaning of “involvement” was perceived as “nebulous” by patients and professionals [29, 30].

We also explored challenges and barriers for carer involvement in inpatient mental health care.

## Methods

### Design of the study

This is a focus group study and data were analysed using thematic analysis. Separate focus groups were held with carers, patients and clinicians in order to ensure that each group could express their views without any concern for dynamics related to interactions with other groups.

### Sampling

Purposive sampling was used for patients as we were interested in hearing the views of both people who were hospitalised at the time of the study and of those currently treated in the community who had a recent experience of inpatient care (less than five years before the study).

For carers and clinicians convenience sampling was used based on self-referral to the study.

Different strategies were employed for recruitment, including asking clinicians to identify patients, advertising the study in inpatient wards at East London NHS Foundation Trust (ELFT), using social media (Twitter) and approaching local carer or patient support groups.

Patients were either identified by inpatient clinicians according to the inclusion criteria or self-referred to participate in the study. Carers self-referred after being approached through carer support groups or seeing adverts on Twitter. Clinicians were recruited through advertising the study in inpatient and outpatient units at ELFT.

Once participants provided assent (patients) or expressed interest for the research (patients, carers and clinicians) they met a researcher in order to discuss the study in detail and provide their written informed consent to participate.

Patients were eligible if they had: sufficient command of English to participate; current or prior experience (within five years) of being hospitalised for psychiatric reasons and at least one friend or family member who supports them with their mental health related needs; and if they were: aged over 18 years and able to provide informed consent for participation in the study.

Carers were eligible if they had: current or prior experience (within five years) of caring for a person who was hospitalised for psychiatric reasons; sufficient command of English to participate; and if they were: aged over 18 years; able to provide informed consent for participation in the study.

Clinicians were eligible if they had current experience of working with patients in a psychiatric inpatient setting.

### Procedures

A topic guide was developed with prompts to encourage participants to generate ideas about the involvement of carers in patients’ treatment in inpatient settings. The topics explored included realities and ideal scenarios of, as well as barriers and facilitators to carer involvement in routine inpatient care.

The groups were facilitated by two researchers of different genders, at least one of whom was clinically trained and able to respond appropriately in case a participant experienced intense distress or become agitated during the group discussion. A lower number of participants was purposively sought when focus groups included patients who were currently hospitalised in order to help them to talk comfortably without overstimulation. These groups included a minimum of three and a maximum of five participants, whilst for other groups a maximum of ten participants was permitted. The maximum duration allowed for a focus group was 90 min. There were no working or personal relationships between participants and researchers.

### Analysis

The focus groups were audio recorded and transcribed, omitting any identifiable information. The transcripts were then analysed using Thematic Analysis [31]. This involved finding common themes in the transcripts and assessing whether there were notable similarities and differences between the participant groups. An interim analysis was performed by JK and AD after the facilitation of nine focus groups. Once 16 focus groups were conducted, an additional meeting of JK, AD and DG took place to discuss the results and decide on whether saturation of the themes had been reached.

The analysis process involved one researcher (JK) coding the transcripts to reflect the content of the text. Related codes were clustered together into themes. Each group of themes was given a label which reflected its content. Each group label was referred to as a “main theme” and the components were referred to as “subthemes”. The second researcher (AD) contributed to the analysis by reading the transcripts and ensuring that no theme is over or under­represented. Any disagreements were discussed iteratively with DG until a decision was reached. NVivo software was used to aid with coding and organising data during qualitative analysis [32].

JK has a background in social sciences and is particularly interested in social and cultural perspectives to mental health. AD is a research psychologist who has carer involvement in inpatient care as her primary interest, in particular with regard to patient views on how this should be implemented. DG is an academic and clinical psychiatrist. His main research interest is acute mental health care and interventions with social networks of people with severe mental illness. These backgrounds may have influenced the personal interpretation of the findings of each author. However, the diversity of the backgrounds and a rigorous methodological approach to analysis with frequent team discussions were used to maximise trustworthiness and helped a balanced interpretation of the material. The different personal views were taken into consideration, but our approach ensured that the interpretation of the findings is not particularly influenced by any of them and was grounded in the data.

## Results

### Participants

Sixteen focus groups were held in 2014 and 2016 which were attended by 86 participants in total (31 patients, 22 carers and 33 clinicians). Five focus groups were held with patients, four with carers, six with mental health clinicians and one with both patients and carers. Attendance per group varied from three to ten participants. Socio-demographic characteristics of the participants have been summarised in Table 1.

**Table 1:** Socio-demographic characteristics of the participants

Gender	Female *n* (%)	Male *n* (%)
Patients	15 (48%)	16 (52%)
Carers	20 (91%)	2 (9%)
Clinicians	17 (52%)	16 (48%)
Age	Mean (standard deviation)	
Patients	43 (12.3)	
Carers	51 (15.8)	
Clinicians	40 (10.4)	
Carer relationship to the patient	N (%)	
Mother	13 (59%)	
Father	1 (5%)	
Wife	2 (9%)	
Partner	1 (5%)	
Sister	2 (9%)	
Daughter	2 (9%)	
Sister and daughter	1 (5%)	
Diagnosis	N (%)	
Psychotic disorders	7 (23%)	
Mood disorders	12 (39%)	
Personality disorders	1 (3%)	
Missing	11 (35%)	
Experience of working in acute mental healthcare in years		
	Mean (standard deviation)	Missing n (%)
Clinicians	9.5 (9.3)	10 (30%)

All patients had experience of admission (either voluntary or involuntary) to a mental health inpatient care within the last five years. Fifteen (48%) of them were females and 16 (52%) were males with the average age of 43 (SD = 12.3). Age was not disclosed from eight patients. Twelve (39%) patients had a diagnosis of mood disorders, seven (23%) had a diagnosis of psychotic disorders, and one (1%) of personality disorder. The diagnosis of eleven (35%) participating patients was not disclosed.

The majority (91%) of 22 carer participants were females who were either a mother (59%, *n* = 13), wife (9%, *n* = 2), sister (9%, *n* = 2), or daughter (9%, *n* = 2) of a patient. One of the participants was both sister and daughter of two people who had been admitted to a mental health inpatient unit within the last five years. Of the remaining two (9%) male carer participants, one was a father and one a partner. The average age of carers was 51 (SD = 15.8) years. Age was not disclosed by seven carers.

Focus groups with mental health clinicians were held for different disciplines separately including psychiatrists, psychologists, qualified nurses, ward managers and support workers. The average length of experience of working in acute mental healthcare was nine and a half years (SD = 9.3) and ranged from two months to 35 years. Seventeen (52%) clinicians were females and 16 (48%) were males with the average age of 40 years (SD = 10.4).

**Fig. 1:**
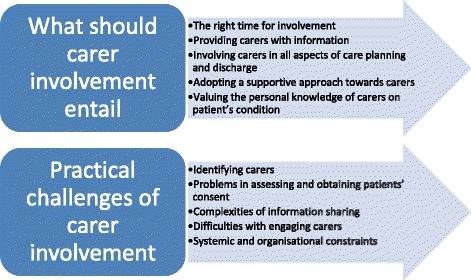
Themes and subthemes

The majority of the themes emerged from all three groups of participant responses, although there were some that were identified only in certain groups (e.g. only mental health clinicians), as it is specified in the description of themes. Participant quotes have been provided to illustrate the themes. Themes are summarised in Fig. 1.

## What should carer involvement entail

### The right time for involvement

Some participants from carer and patient focus groups felt strongly about involving carers as soon as it is possible and ideally within the first few days from the patient’s admission in order to provide immediate support and comfort for the patient and ensure that the carer’s view is taken into account.

*“That’s where the carers come in, doesn’t it? Initially the first, second, third day, that’s where the carer’s voice should be heard more than being pushed aside.”* (Carer - C17)

*“I think straight away or even within a day because it’s very important when someone’s unwell for them to be, you know, comforted and supported from their families and loved ones”* (Patient - SU20)

However, others preferred carer involvement to happen later after admission. Clinicians emphasised the importance of initial admission procedures that they needed to prioritise. A view was expressed that although early involvement would be the ideal scenario, the right timeframe for carer involvement may vary depending on the patient and their clinical presentations. This was seconded by some patients and carers.

*“I think there are more important things to deal with in the meantime, like especially if a patient comes in really unwell, and say they're secluded or something.”* (Clinician - P32)

*“It takes me at least a week proper week to realise everything. I don’t realise where I am, I don’t realise nobody, I can’t remember the doctors’ faces.”* (Patient - SU22)

*“They could be dosed up and completely out of it. They might be still deluded or whatever you know (C5: not settled), yeah, and till they’ve really set to realise where they are… because often they don’t realise where they’re until they’ve had medication and a bit of therapy or whatever.”* (Carer - C1)

### Providing carers with information

The provision of information was generally seen as an important element of carer involvement in treatment. Patients and carers felt particularly strongly about it. Patients talked about the provision of information as a way of educating carers to better understand the realities of patients and adopting a more supportive and less judgemental approach towards them at home.

*“I wish my parents had a better understanding about my illness (SU10: same here) and my family around this ‘cause they have no insight about it at all (SU8: no). They think it’s just (SU8: you playing up). You playing up (SU8: mmm) (SU10: attention seeking) yeah.”* (Patient - SU9)

*“I think they should they, the carers, should have more education on mental health, so that they don't judge you or-or treat you differently.” *(Patient - SU30)

Carers also expressed the wish to equip themselves with information and reported occasions when the lack of it caused them feelings of frustration, worry and being left alone or “in the dark”. Clinicians supported this view and regarded information as having a reassuring effect on carers and helping them to take care of their relative or friend at home.

*“It’s really important for me I’ve been very frustrated, you know, ripped apart in a way because I had no information.”* (Carer - C12)

*“When I went on the ward I didn’t know anything. You’re just left in the dark. Nobody talks to you. It just felt isolated and lonely. You’re so worried about your loved one who is not all there and you want to get all the information.”* (Carer - C17)

*“I think it reduces the stress of the carers as well, if you get it right, because I think a lot of the time they can feel a bit pushed out of things, like ‘This is the medication your son or daughter is taking.’ That's it, you know? And they come with all of these worries and they're not always addressed, so I think when they are, it helps them as much as it helps the patient.”* (Clinician - P32)

Information on psychiatric medication and its side effects was given a particular importance by participants. Patients felt that their behaviour might be interpreted differently by those who do not have an understanding of side effects, which may potentially damage their relationship with carers. Carers wished not only to know what to expect from medication in terms of side effects but also wanted to understand the decision making process that had gone into it.

*“They should be informed on what medication you're on and its possible side effects. Because I mean you know you can have some side effects where it might make you a little bit aggressive demeanour or something and they can take it personally and then they don't want to care for you, you know what I mean?* (Patient – SU30)

*“… why they’ve chosen it [the medication] over others ‘cause there are so many different ones they obviously have to choose from <…> so we know what the decision making was that was involved in that (C11: also possible side effects so you know what to look out for).”* (Carer - C15)

In addition to the information on mental illness and medication, carers wished to be provided with regular updates about the service users’ progress and daily activities while in hospital, including when they get transferred or discharged from the ward. Furthermore, they felt that inpatient procedures, different roles of the multidisciplinary team and carers’ legal rights should be explained to them when their relative or friend is admitted on a psychiatric ward.

*“I think also it’s quite good when you get feedback on the things that they’ve been involved when they’ve been in hospital. Another thing that would be helpful I think knowing who the people (professionals) are, their names, their roles, what their job is.”* (Carer - C11)

*“My child was transferred to [hospital] without my knowledge although that very day they had a ward round nobody told me that (C3: mm) um he was going to be transferred (C3: yeah, it happened to me) to another hospital.”* (Carer – C5)

*“Mental Health Act - I haven’t got a clue what that’s all about. I know there are a lot of acts there. I don’t know my rights or my wrong, and tribunal, and all that lark. So those things, not being told…just have to go and Google it myself and educate myself. That’s what I’ve been doing all this time is educating myself.”* (Carer - C17)

### Involving carers in all the aspects of care planning and discharge

All three participant groups highlighted the importance of fully involving carers in the process of care planning and discharge. Carers felt they should be involved in everything while their relative or friend is in the inpatient unit. Attending ward rounds was regarded as one of the main ways for carers to provide their input. Patients expressed that carer involvement in discharge planning would contribute to their quality of care in the community.

*“At the end of the day I’d like to be involved in everything ‘cause s/he’s not in my care, s/he’s not at home, s/he’s in a hospital.”* (Carer – C2)

*“So of course they need to be a major part of ward round. They need to be told what is happening, what’s the next step where we’re going from here, they have to be part of the discharge planning, they need to know exactly everything that’s happening with the patients.”* (Clinician – P17)

*“I think the assessment that you have prior to being released, I think it's a good point for them to get involved (Mod2: discharge planning?). That's right, ‘cause then they know about the medication, they know about your moods and it just gives them an idea of when you come out, what they're gonna have to... (SU26: deal with) Deal with, yeah.”* (Patient - SU28)

The discussions about the involvement in care planning often involved emotional reflections of previous carer experiences with mental health services that involved lack of communication or excluded them from having a more active role in decision making.

*“There’s a ward round coming up.’ I get a phone call from [ward name] saying, ‘Can you be in at…’. I’m not saying it happens all the time, but it has happened. It’s been left to the last minute. By the time I’ve got a bus up here and I’ve got myself ready, it’s on its way and s/he’s been done and dealt with.”* (Carer - C21)

### Adopting a supportive approach towards carers

Participants reflected on the impact that looking after someone with mental health difficulties has on carers, who often report being emotionally and physically exhausted as well as frightened and worried for their relative or friend who gets admitted to an inpatient psychiatric unit. All three groups acknowledged this and suggested that carer involvement should include a supportive and reassuring approach towards those who dedicate their time and energy to caring for a relative or friend. Carers and clinicians suggested that the supportive approach should encompass empathy, reassurance and validation of carer experiences.

*“It’s scary when they go in and they’re really unwell and what I found very helpful was very calm staff that are really reassuring and if you could have that at that stage in a meeting like that, I think that would help everybody’s anxiety.”* (Carer - C11)

*“Some work around validation of their experiences is extremely important as a starting point” *(Clinician – P6)

All three participant groups emphasised the importance of staff asking carers how they are doing and if they need support when their relative or a friend gets admitted to an inpatient unit. However, it was also pointed out that sometimes it is not straightforward to identify a carer and carers’ needs cannot be assumed but an individualised conversation is required to explore them.

*“I was actually asked how I was by the staff on the ward and that was new experience, really positive thing. Generally concerned and interested, which was lovely.”* (Carer – C11)

*“It would be nice for perhaps her/him to... (SU26: get...) get some support (SU26: yeah). Even if it's once a year, you know just somebody to just see how s/he is.”* (Patient - SU28)

*“There’s always an assumption that, “oh they’ve got a carer, we must do a carer’s assessment. Oh they’ve got a carer, we must offer them some support”. I’m much more minded to say, well, if you are carer, do you want support? Let’s have a discussion rather than just automatically assume you must have it” *(Clinician - P28)

### Valuing the personal knowledge of carers on patient’s condition

All three participant groups felt that carers should be respected as experts in their relative or friend’s condition as this would make it more likely that their involvement in care takes place and is helpful for assessment and care planning.

*“The knowledge the carer brings to that, I think, including someone in that sort of decision making and care planning has to acknowledge that they bring knowledge and expertise themselves.”* (Clinician - P12)

*“They know you, they know your likes, your dislikes, your triggers. They’ve been around you so they’ve gone through the process of you going from being well to getting unwell, so they they’re kind of experts around your care.” *(Patient – SU7)

Carers in particular felt strongly about their knowledge being recognised and their voice being heard when decisions are made about the patient’s care in the inpatient unit. They recounted previous experiences and emphasised the importance of clinicians taking their views into account.

*“Sometimes you don’t have the chance to tell them what’s your view, what’s happening and they don’t want to listen to you. They just put you back in the background *(C11: yeah). They just tell you to shush, you know.” (Carer – C12)

*“I’ve had recent experience where I have been listened to and it was fantastic the impact on my daughter because the care was so good and the way I was listened to was huge – she got better quicker.” *(Carer – C11)

## Practical challenges of carer involvement

### Identifying carers

Clinicians reported that they often found it challenging to identify who the carer was when someone is admitted on a psychiatric ward. They cautioned against staff making assumptions about who the person’s carer might be and felt that they should always consult patients first and be helped by them in defining who the carer is and how s/he needs to be involved.

*“If you’ve got somebody coming on the ward with a bunch of flowers to just say ‘hi’ to that patient we’ve got on the ward, we are not going to assume they are a carer unless the patient, service user or client says it, do you know what I mean? I don’t think we assume that they are a carer. A service user defines who is their carer as opposed to just us. We are going to have to be guided by them.”* (Clinician - P28)

*“First and foremost is to speak to the service user and who they identify. Because you’d be surprised sometimes, it’s not the immediate family, sometimes it’s not even their partner.”* (Clinician – P22)

However, asking patients about their supportive relatives or friends was not always possible in the acute inpatient mental health settings when persons just admitted to an inpatient unit were not well enough to express their wishes. This was highlighted by both patients and clinicians. In situations like this, clinicians would try to identify carers from the context by observing supportive people who come to visit the service user or where with the person at the time of admission.

*“I think if the person like I was really unwell so how would I actually identify who I want to be involved in my care? How would I be able to voice… because I was so unwell I didn’t know…I was in a psychotic state so how would I be able to inform you that I want so and so involved?”* (Patient – SU7)

*“Some of it is inferred by generally the people who have turned up when the patient is admitted, the people that come to the ward and visit the person.” *(Clinician – P28)

Some clinicians also emphasised their need for vigilance when identifying carers as there may be concerns for exploitation of the patient.

*“Then also at the same time we need to be very very, you know, vigilant to be sure that this so called carer is not asked to take any benefit or in any way abuse the vulnerability of the… of the patient because some, you know how people can take advantage of people” *(Clinician – P19)

### Problems in assessing and obtaining patients’ consent

Obtaining consent for carer involvement was another challenge for clinicians working in acute mental healthcare settings. It was pointed out that at times patients would not give their consent to involve their relatives or friends. A number of reasons for this was provided by patients such as the wish to protect their relatives and friends from distress and worry, problematic relationships with their family, including pressure and over-involvement, as well as stigma and embarrassment.

*“I don’t want them knowing everything that I do because it just hurts them.”* (Patient – SU8)

*“I don’t have a very good relationship with my family at the moment. I think they intervene too much.* (Patient – SU9)

*“I didn't involve anyone at all, and that was through embarrassment <…> (SU25: stigma, isn’t it?)”* (Patient – SU24)

Participants from all the groups expressed that patients have the right not to provide consent for carer involvement and that this right should be respected.

*“But they have the choices, and I do respect the choices if they don’t want us to be involved. You have to have a bit of respect for them, even though they’re not well and they’re ill, you still have to sort of respect the choices and listen to what they’re saying.”* (Carer – C17)

*“It's their choice and their rights.”* (Clinician – P33)

*“I think it should be the individual that decides and the choice should be given to them.” *(Patient – SU28)

However, at the same time, all three participant groups reflected on the patient’s capacity to consent and on the possible occurrence of specific symptom features (e.g. paranoid delusions) or difficult relationships before admission. All these issues may influence the consent process. Carers often favoured advance statements, which in their view provided some kind of guarantee that they will have a say in the patient’s care.

*“I suppose the obvious one is that due to the health condition, they might be too distressed or lack the capacity to make the decisions.”* (Clinician – P24)

*“Let’s not forget. Let’s say, at the time of admission they are disorientated. My son/daughter, s/he blames me for going to hospital. So if you ask her/him, s/he’s going to say, ‘I don’t want my mother involved’.”* (Carer – C19)

*“You can make a plan when they’re well so they know well actually I do want these carers involved but when I’m unwell I might become… I might say no to this but you know to actually discuss it when they’re well.” *(Carer – C11)

### Complexities of information sharing

Patients felt strongly that they should be able to specify what personal information they wish to remain confidential and what information they would like to share with their carers. Previous experiences were recounted by patients when their preferences for information sharing were not followed by staff.

*“I came with an ambulance to A&E psychiatric with my partner and since I couldn’t answer the questions the psychiatrist was talking to my partner and s/he said, “but are you aware that s/he committed suicide in [year]?”, and it’s not something that I especially wanted to share.* (Patient – SU12)

All three groups of participants agreed that patients should be offered the option of being present in the room when personal information is being shared with their carers. This would provide them with an opportunity to express if they felt that the information was incorrect. While some patients felt that they should always be present and be involved in discussions about them, others preferred not to listen to these as this would cause them distress.

*“If someone was talking about me, I'd like to know what they're saying. And as I say I - if you know someone's talking about you, what are they saying? And then, that can also make you- (SU25: - paranoid) yeah, revert back *(Patient - SU24)

*“I probably would get angry and frustrated with them talking about me in the room. I couldn’t bear that.”* (Patient - SU8)

However, it was also pointed out by clinicians that having the patient in the room when they are sharing information with the carer may not always be possible. In these cases, clinicians felt that the patient should be informed what information was shared with their relative or friend. This approach ensuring transparency was supported by patients.

*“I’d personally probably go back to the service user and say, “I spoke to your mum about that and that”, so involve them as much as possible (P13: mm) ‘cause you’re trying to re-connect the families establish communication and it wouldn’t be helpful if you did something behind their back.”* (Clinician – P10)

*“As long as they are aware of that’s happening I think as well. They don’t necessarily have to take part but are kept in the loop sort of thing (SU12: Yeah, otherwise, like they’re talking behind your back).”* (Patient - SU15)

Some carers raised their concerns about feeling under pressure when sharing information in the same room with their relative or friend and preferred to give information in a separate meeting.

*“It is a delicate one because my son/daughter used to… s/he’s in the room and s/he’s like, oh God I’m not a nurse or anything, s/he might penalise me, you know… I might get into trouble.”* (Carer – C2)

Furthermore, carers felt that more helpful communication should be ensured when the patient does not provide consent for personal information to be shared with them. Clinicians admitted that their communication with carers in these situations could be improved. Experiences of a less than emphatic pattern of communication were reported by carers and clinicians.

*“We had to call hospital before and we’ve asked how is s/he, “sorry, due to data protection we can’t actually tell you”. Are you joking?! This is my sister/brother! You can’t tell me how s/he is? “We can confirm that s/he’s present in our ward”, you know, that kind of robotic response. It’s not helpful when you’re already going through trauma yourself and then being told they can’t tell you how s/he’s doing.”* (Carer – C15)

*“I think we need to think about the way we speak to the family members when the patient doesn’t want them involved in the care because I’ve seen people on the phone like staff talking to relatives and stuff in a way that don’t know just… (P8: they just don’t give away too much). Yeah, “sorry we can’t give information” and we’re not very helpful and it’s we’ve got to understand that these people are really worried about their family, so we’ve got to try and find that balance.”* (Clinician – P7)

When attempting to facilitate carer involvement, clinicians often found themselves in a difficult situation trying to accommodate both patients’ and carers’ needs and finding the balance between their wishes.

*“I think sometimes you find that when someone's quite unwell, often they don't want their family or carers involved, which makes it really difficult when the family do want to be involved, and then you're limited in how much you can actually involve them because you have to respect the wishes of the service user. And that can be really hard, because that's really upsetting for the carer. And then you have to try and find a way to balance both needs, and that's really hard.”* (Clinician - P31)

### Difficulties with engaging carers

Clinicians and patients pointed out that at times it might be difficult to access carers. Carers fulfil their supportive role for patients, whilst often also having to deal with other aspects of their life such as work and other family commitments. This may make carer involvement in care in inpatient care difficult, especially if the inpatient unit is not located close to carers’ homes.

*“If they’re working, maybe, you know, they might have other priorities, you know, they have to follow their job role, emergencies. Sometimes it could be, you know, loads of things - they’re busy, they’ve got children, they can’t get childcare, so lots of stuff.” *(Patient - SU20)

*“If they have relatives, like I said, up north, they are not going to come down for this meeting.” *(Clinician - P29)

Cultural and language barriers were also described by both clinicians and patients as contributing to difficulties in involving carers. Clinicians focused on practical challenges such as getting interpreters for meetings with carers. Patients critically reflected on the diversity of understandings about mental health in different cultural communities.

*“Language barriers, especially like [language] patients when you don't understand and their mum's a bit… I've had a few mums that get quite annoyed that I don't understand what they're saying, and it helps when you do have a [language] speaking person on the ward. That does help. But other times it is a bit… you're trying to communicate something to somebody that you can't always get across, and you can see that anxiety.” *(Clinician - P32)

*“We've got so many different communities different cultures and different understanding about mental health. Say for example, somebody is not happy with having an arranged marriage and that is causing a lot of their problems. For them to sit down is going to be difficult. And then getting people from different communities to talk, you know?”* (Patient - SU28)

### Systemic and organisational constraints

Clinicians, in particular, pointed out that the lack of staffing and time on the ward as a most important organisational constraint that impacted on their attempts to involve carers. While this was not extensively discussed by other groups of participants, these concerns were also raised in a carer focus group.

*“Once you're on the ward you get sucked in (P30: That's it.) to everything that's going on. It's really difficult (P30: yeah) to get away.”* (Clinician - P32)

*“Quite often we may be too stretched or busy to even be thinking on that level, ‘cause we're just, you know, running through sort of tasks” *(Clinician – P30)

*“Overall, I don’t know, is there difficulty with them in terms of staff level, staff numbers, that they don’t have somebody to, obviously, attend to us? I don’t know.”* (Carer – C19)

Clinicians also critically reflected that often it was unclear whose responsibility carer involvement was. However, at the same, they cautioned against having just one person responsible and felt that the whole team approach should be adopted.

*“I think there's a lack of clarity as to whose role it is. I mean, on some wards there's an allocated nurse who is the carers' specialist if you like, who will run a programme and sort of arrange meetings. So, if that isn't in place, and other members of the team aren't clear on who, where, what, when.” *(Clinician - P30)

*“You can't just have one person put in charge of this. That's horrible to be like the only person put in charge of something”* (Clinician – P32)

Furthermore, clinicians described the current inpatient mental health system as lacking flexibility in terms of including carers in care planning and accommodating their needs. They felt that they could provide a better service for carers and involve them more if the system were more carer-friendly.

*“We could provide a service… if it was more flexible (Several: yeah).” *(Clinician – P26)

*“I think it’s lack of accessibility for them to us. Services aren’t necessarily geared up to be the most supportive to carers. It’s not flexible enough for a carer to access help.”* (Clinician - P28)

Finally, clinicians suggested that working with carers requires effective communication and facilitation skills in managing often complex family dynamics and different needs. It was felt that training on working with carers should be provided to staff who will be involved in this role.

*“And train them quite a bit on this I think ‘cause you may have to meet a potentially complex patient or complex family.”* (Clinician – P1)

*“You have to be quite skilled to do that because it’s very easy to get involved in rather very difficult conversation where the family will be offloading their distress and their anger and so forth. So if you meet with all of them, you have to handle that in some ways and it is maybe very difficult.”* (Clinician - P5)

## Discussion

### Main findings

Our approach identified themes that are shared by patients, carers and clinicians. This builds on and complements existing literature, guidelines and training into carer involvement, which has largely been professional-led. The inclusion of patients and carers helped to specify the barriers and components that are especially helpful to improve their experience of care.

Many participants felt strongly that carer involvement should happen as early as possible following admission. Yet, others mentioned that this may be difficult for some patients, who are particularly agitated or lack capacity to consent. To participants, “involvement” meant first and foremost the provision of information to carers but also participation in all the decisions about treatment. There was emphasis that the carers’ personal knowledge of patient’s condition should be acknowledged in order to empower them to contribute actively to decisions about care. On the other hand, the importance of providing emotional support to carers was also pointed out, especially during the difficult and stressful time of a relative’s or friend’s admission to an inpatient unit.

Practical challenges for carer involvement were about identifying carers during an acute crisis, establishing prompt and appropriate procedures for assessing the consent of patients and determining which information can be shared with carers. The sub-theme ‘systemic and organisational constraints’, unlike others which were shared among the three different groups, was more specifically put forward by clinicians. Contacting and identifying carers during a crisis and engaging with people from different cultural backgrounds and with different views about mental illness are commonly encountered difficulties. A whole team approach was favoured above solely training individual staff members. Participants felt that all the members of the clinical team should facilitate and/or cooperate in the work with carers.

### Strengths and limitations

To our knowledge, this is the first study to explore carer and patient views on how to maximise carer involvement in inpatient mental health care. We included a wide range of patients with different diagnoses and a large number of participants with current or recent experience of inpatient care.

However, this study had the following limitations which need to be taken into account. The participants were recruited in a specific geographic area (East London) which is an urban area in rapid demographic expansion and consequent high pressure on psychiatric beds. This might impact on how applicable the results are to other areas. Patients or carers who are more engaged with mental health services may have also been more likely to participate in research. Moreover, focus groups are powerful in generating views but less sensitive in capturing experiences; however, our methodological choice was guided by the aim to generate views on what are the components and potential challenges of carer involvement in inpatient care. Finally, due to the nature of focus groups, involving spontaneous and responsive verbal interactions between different people, only people who had conversational English could participate.

### Comparison with the literature

A previous study including only carers had identified logistic issues, in particular distance from the services, different cultural understanding of mental illness and problems with confidentiality as potential barriers to carer involvement in mental health care [22]. In the current study, we had a closer look at carer involvement in inpatient treatment identifying additional challenges and how carer views were reflected by other parties, such as patients and clinicians.

Many participants felt that carers should be involved in inpatient treatment as soon as possible after admission, although challenges related to a very acute clinical presentation of the patients and to the capacity of the patients to make decisions in some clinical states were pointed out. At least for some patients, prompt involvement of carers might be a mediator of these good outcomes facilitating adherence and engagement with care [4, 33] as well as the identification of early warning signs of relapse which may prevent hospitalisation [3].

Problems with obtaining consent from patients and difficulties in establishing what type of information should be shared can hinder or delay carer involvement in inpatient care. Patients may be unable (because they lack capacity) or unwilling to consent to carer involvement soon after admission. It is important to respect individual autonomy including the freedom to make one’s own choices, and independence of patients [34].

However, lack of carer involvement may also be caused in some cases by lack of structured procedures for identifying and contacting carers, which is something that may be prevented. This may cause perceptions in carers that staff do not have enough concern for the value of carers’ role [35]. Withholding information is also seen by carers as having potentially negative consequences especially after discharge [20, 35].

Difficulties in communicating with families may arise from lack of training and supervision of staff in delivering interventions or sessions to both patients and carers [26, 28, 36]. These meetings may be challenging because of previous conflicts between families and staff at the point of initial help seeking [37]. The need to deal with different cultural or personal views about mental health and mental illness also emerged in our findings as a potential reason for problems in engaging families and could delay or make it difficult a collaborative arrangement of care for people from specific groups [38].

There was a recurring theme that carer involvement should be a shared mission of the entire clinical team in order to facilitate its implementation. Many ward staff members work shifts, hence training or supporting just few members of the clinical team to work with carers is a strategy that rarely pays off [26, 39]. On the other hand, it may be unfeasible to train all staff to deliver complex carer-oriented interventions. Guidelines suggest that all mental health services need to inform and involve carers in treatment [1]; however, the exact procedures that need to be followed are decided on a local basis with varying degrees of implementation of the good practice standards [2].

### Implications

Difficulties in identifying who the carers are and in contacting them for sessions are important barriers to carer involvement in inpatient psychiatric treatment. Structured procedures which can be carried out by all or most members of staff may help with ensuring that carer can be informed and involved in decisions as soon as possible. In case they are not able to commute to the ward, online communication technologies such as video-conferencing (for example, Skype) may facilitate contact with patients and clinicians on the ward. These procedures may facilitate the early involvement of carers, at least when it is held back by logistic problems.

In some cases, patients may lack capacity to consent to a session with carers or refuse for a number of reasons, related to their mental state or not. It is important in these cases to make sure that appropriate information is provided to patients both on the benefits of carer involvement for mental health treatment as well as their rights to refuse sharing of particular information, so that their decision can be appropriately supported.

Once carers are able to participate in sessions, it is important that clinicians are able to communicate effectively in order to express respect and empathy. Communication skills training packages for mental health clinicians have already been successfully tested [40] and could be adapted in order to incorporate the additional difficulties related to facilitating a three-way conversation, involving not only patients but also carers. Cultural sensitivity is also an important issue and, in increasingly multicultural services, clinicians need to be able to understand and discuss views from different cultural backgrounds and belief systems. This will help not only to involve carers but also to set up appropriate and effective care plans with patients from minority groups [38, 41, 42].

The barriers reported emphasise the need to develop simple and structured procedures that are easy to implement for all clinicians and do not require too much time or resources. Identifying and contacting carers, providing information and involving carers in decisions and care plans seem to be the most important aims of these procedures.

## Conclusions

The findings of this study, integrating the results of the previous literature [22, 28–30] as well as the views of different parties involved, and focusing specifically on inpatient mental health care can help formulate a framework for good practice in carer involvement in inpatient care.

**Fig. 2:**
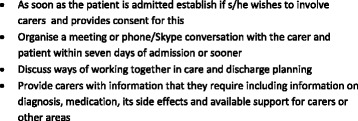
Protocol for carer involvement in inpatient mental health care

A crucial aspect of this is that we asked participants to identify what carer involvement should entail, which was a knowledge gap reported by the previous literature [29, 30]. A framework for carer involvement in inpatient psychiatric care, resulting from our findings is reported in Fig. 2.

Simple interventions that intend to meet all these aims need to be developed and tested in real-life contexts. The evaluation of these interventions should explore whether they facilitate the involvement of carers in the inpatient treatment of a higher number of patients and more positive outcomes for them. It is also important to capture the individual experiences of participating patients and carers in order to further tailor these interventions to their preferences. Studies looking at differences related to patient characteristics (for example diagnoses) may also help to elucidate more specific aspects and challenges for carer involvement. Finally, involving patients and carers as active participants of research, contributing to the process of design and analysis might help to enrich the quality of subjective experiences captured.
